# Sources of Variability in Musculo-Articular Stiffness Measurement

**DOI:** 10.1371/journal.pone.0063719

**Published:** 2013-05-08

**Authors:** Massimiliano Ditroilo, Mark Watsford, Aron Murphy, Giuseppe De Vito

**Affiliations:** 1 School of Public Health, Physiotherapy and Population Science, University College Dublin, Dublin, Ireland; 2 Department of Sport, Health and Exercise Science, Faculty of Science, University of Hull, Hull, United Kingdom; 3 Sport and Exercise Group, UTS: Health, University of Technology, Sydney, Australia; 4 Institute for Sport and Health, University College Dublin, Dublin, Ireland; Ohio State University, United States of America

## Abstract

The assessment of musculo-articular stiffness (MAS) with the free-oscillation technique is a popular method with a variety of applications. This study examined the sources of variability (load applied and frequency of oscillation) when MAS is assessed.

Over two testing occasions, 14 healthy men (27.7±5.2 yr, 1.82±0.04 m, 79.5±8.4 kg) were measured for isometric maximum voluntary contraction and MAS of the knee flexors using submaximal loads relative to the individual's maximum voluntary contraction (MAS_%MVC_) and a single absolute load (MAS_ABS_).

As assessment load increased, MAS_%MVC_ (coefficient of variation (CV)  =  8.1–12.1%; standard error of measurement (SEM)  =  51.6–98.8 Nm^−1^) and frequency (CV  =  4.8–7.0%; SEM  =  0.060–0.075 s^−1^) variability increased consequently. Further, similar levels of variability arising from load (CV  =  6.7%) and frequency (CV  =  4.8–7.0%) contributed to the overall MAS_%MVC_ variability. The single absolute load condition yielded better reliability scores for MAS_ABS_ (CV  =  6.5%; SEM  =  40.2 Nm^−1^) and frequency (CV  =  3.3%; SEM  =  0.039 s^−1^).

Low and constant loads for MAS assessment, which are particularly relevant in the clinical setting, exhibited superior reliability compared to higher loads expressed as a percentage of maximum voluntary contraction, which are more suitable for sporting situations. Appropriate sample size and minimum detectable change can therefore be determined when prospective studies are carried out.

## Introduction

Musculo-articular stiffness (MAS) measured with the free-oscillation technique is a comprehensive measurement of joint stiffness which includes the stiffness of the muscle-tendon unit, skin, ligaments and articular capsule, along with a number of other mechanical and neuromuscular factors [1]. The assessment of MAS has implications for muscular performance, injury occurrence and gender differences [1]. The construct validity of the free-oscillation technique has been ascertained with a positive linear relationship between MAS and rate of torque development [2] and a negative relationship between MAS and either electromechanical delay [3] or performance augmentation (as a result of the pre-stretch action) [4]. Reliability of the method has also been established in a number of papers, with an overall level of absolute reliability higher than the relative reliability [1].

When assessing MAS with the free-oscillation technique, the joint is modelled as a single-degree of freedom spring-mass system with a damping element, with an assumption of linearity of the model [5], [6]. Despite some evidence of nonlinearity of the damped acceleration signal [7], the vast majority of studies using the free-oscillation technique adopted a linear model, which is easier to use and has been granted construct validity [1]. The stiffness value is obtained as follows [4], [6]:
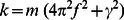
(eq.1)


where k is the MAS (N·m^−1^), m is the load supported (kg), f is the damped natural frequency (s^−1^) and γ is the coefficient of damping (s^−1^). It was previously demonstrated that γ is negligible as it contributes less than 1% to the total stiffness value [8]. Therefore, eq. 1 can be approximated as:
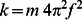
(eq.2)


Accordingly, k varies linearly with mass and exponentially with frequency variation.

While frequency is a measured variable which depends on the elastic characteristics of the structure assessed [9], mass is the load added, along with the weight of the body segment under analysis. Typically, either fixed loads or multiple loads expressed as a percentage of maximal voluntary contraction (MVC) are utilised with the aim of reproducing the loads supported during functional activities [3]. Specifically, if an investigation applies a repeated-measures design, absolute assessment loads may be appropriate [10]. Conversely, when comparing MAS between individuals, relative loads must be used to prevent bias due to differences in mass and strength, as occurs when comparing males and females [8], subjects of different body mass [11] or athletes of different levels [2].

By virtue of equation 2, it is evident that a component of any change in stiffness originates from a change in frequency, while an additional element comes from the variability in MVC assessment and its consequent use in determining the submaximal load. The latter is exacerbated when MAS is measured prior to and following an intervention which has probably altered the level of MVC [12], [13].

Whilst two studies have identified the issue of a dual source of error [3], [14], the proportional contribution of each component to the overall MAS variability at varying loads has yet to be established. Accordingly, the aim of this study was to determine and quantify the sources of variance when MAS is assessed with multiple loads relative to MVC, or a constant absolute load.

## Methods

Fourteen men (27.7±5.2 yr, 1.82±0.04 m, 79.5±8.4 kg), physically active but not involved in competitive sport, volunteered to participate in this study. They gave written informed consent and avoided any strenuous physical activity 24 hours prior to each testing session. Ethical clearance was granted from the Ethical Committee of University College Dublin (Ireland). Two series of tests, separated by at least 2 weeks, were carried out and administered randomly, both requiring 2 sessions within 7 days. Participants were tested for MVC of the knee flexors (KF) and MAS using submaximal loads relative to the individual's MVC (MAS_%MVC_) over two sessions. In a further two sessions the participants were tested for MAS using a single, absolute load (MAS_ABS_). MAS of the KF has been commonly assessed due to its relationship with locomotion [6] and injury [15]. Only the right leg was considered in all testing sessions which were preceded by a standardized warm-up.

**Figure 1:**
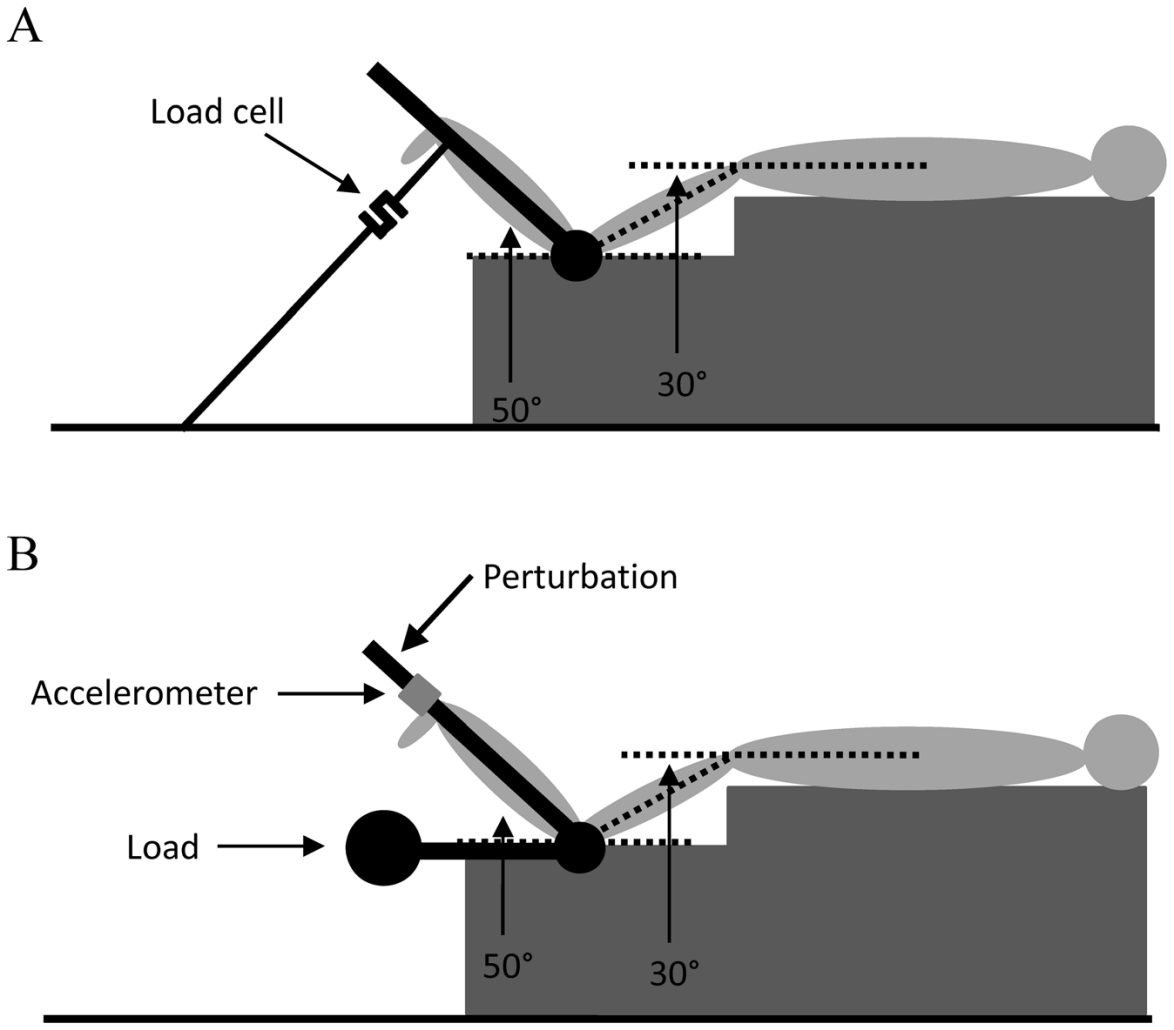
Schematic diagram of the position used to assess knee flexor maximal voluntary contraction (A) and musculo-articular stiffness (B).

Isometric MVC of the KF was measured with participants positioned prone on a padded table, firmly strapped at the hip, with the thighs supported in 30° of hip flexion below the horizontal and the knee flexed at 50° above the horizontal with a knee angle of 100° [15]. A custom-made leg-curl machine, equipped with a load cell (Leane International, Parma, Italy, measurement range: 0–500 kg, output: 2.00 mV/V), was used for the test described (figure 1A). The subjects were required to exert a force against the dynamometer lever as strongly and as quickly as possible for 3–4 seconds while verbal encouragement was given. They underwent a number of trials until 2 results were obtained which did not differ by more than 5%. Data was low-pass filtered (fourth order, zero lag Butterworth filter, cut-off frequency 15 Hz) and the trial with the highest peak force was used for later analysis. MVC (N) was determined as the trial with the highest peak force with the addition of the weight of the limb [16] and the weight of the lever of the dynamometer.

Using the same machine and set-up described above, KF MAS was measured with a free-oscillation technique. The participant's leg, which was thoroughly strapped to the lever arm of the machine, supported the load on the distal portion of the lower leg, approximately 2 cm superior to the lateral malleolus, with the knee flexed at the correct angle. The participant was instructed to hold the load for approximately 3–5 s, with the foot in neutral position (0 deg at the ankle), during which a downward manual perturbation in the order of 100–150 N was applied perpendicular to the distal end of the lever arm. The ensuing damped oscillations were recorded by means of an accelerometer (Crossbow Technology, Milpitas, California, USA) attached immediately proximal to the point where the perturbation was applied (figure 1B). After thorough familiarization, where the participant was instructed not to react to the perturbation, but to focus their attention on keeping the same level of contraction throughout, four technically correct trials were recorded for each load. The average of them was calculated, so that one result for each variable was obtained and used for later analysis. The absence of bursts of electrical activity of the muscles (measured via surface electromyography of biceps femoris), along with the presence of damped oscillations were used to assist in judging an acceptable trial.

The acceleration signals were filtered using a fourth order Butterworth filter with a cut-off frequency of 4 Hz, and the frequency of the first cycle of oscillations was determined along with the average frequency of the four trials, which was considered for later analysis. For the two sessions of MAS_%MVC_ assessment, loads of 15, 30, 45, 60% of the specific MVC measured on each day were used in a non-randomized order. At the two constant load sessions to assess MAS_ABS_, a load of 6.5 kg was applied as described above. This load corresponded to approximately 35% of MVC.

Results are expressed as mean ± SD. Coefficient of variation (CV) of load, frequency and MAS variables were calculated as:
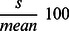
(eq.3)


where s is the standard deviation and mean is the mean of the two results obtained on testing session 1 and 2. Standard error of measurement (SEM) was also calculated as 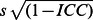
(eq.4)


where s is the square root of the total sum of squares divided by ‘number of observations - 1’ (as in an ANOVA analysis) and ICC is the intraclass correlation coefficient. Confidence limits of CV and SEM were also determined [17], [18]. Repeated measure ANOVA was used to examine the difference between CVs obtained at various loads (15, 30, 45, 60% of MVC and constant load) for two dependent variables (frequency and MAS). When a significant effect was found, the post-hoc Tukey's method was used to identify where significant differences lay.

Systematic bias between testing sessions was analyzed using a paired t-test and an alpha level of p<0.05 was considered statistically significant for all tests.

The calculations related to the propagation of errors (uncertainties that attend all measurements) [19] were used to assess the theoretical specific contribution of variation related to mass (CV_m_) and frequency (CV_f_) to the overall MAS variation (CV_MAS_). With reference to equation 2, the error related to k, m and f are δk, δm and δf, respectively, whereas there is no error associated to 4π^2^. Since δm and δf are independent from each other, δk can be estimated as follows [19]:
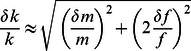
(eq.5)


As δk/k, δm/m, and δf/f are percentage errors, CV can be substituted into equation 5 to obtain:
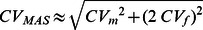
(eq.6)


The statistical analysis was performed using Microsoft® Office Excel® 2007 and Statistica software, version 9.1 (StatSoft LTD, Bedford, UK)

## Results

The mean ± SD of MVC for KF was 180.6 ± 42.7 and 192.1 ± 49.9 N on testing session 1 and 2, respectively, with no significant difference (p = 0.15). No systematic bias was detected (p>0.05) in any of the variables considered. The results of load, frequency and MAS from the two testing sessions, along with the reliability coefficients, are summarized in tables 1, 2 and 3, respectively. Notably, while CV was constant for the load variable (6.7%), MAS (table 3) and frequency (table 2) CV increased as load increased, however the ANOVA post-hoc analysis did not show a significant difference (p = 0.15 to 0.44). In contrast, when a constant load was used, CV was considerably lower in MAS (table 3) and frequency (table 2) variables compared to multiple loads and the post-hoc analysis revealed to be significant different from MAS_MVC60%_ (6.5 vs 12.6% and 3.3 vs 7.0%, respectively; p<0.05). A similar pattern was observed for SEM, which increased as load increased for all variables, though when a constant load was used it was reduced, on average, by 35% (0.039 vs 0.060 s^−1^) to 48% (0.039 vs 0.075 s^−1^) (frequency, table 2) and by 22% (40.2 vs 51.6 Nm^−1^) to 59% (40.2 vs 98.8 Nm^−1^) (MAS, table 3).

**Table 1:** Summary of the loads corresponding to different percentages of maximal voluntary contraction, along with a constant load of 6.5 kg, adopted during the two testing sessions.

	Mean (SD) (kg)		Reliability coefficients			
	T1	T2	CV (%)	95% CL	SEM (kg)	95% CL
MAS_MVC15%_	2.76 (0.65)	2.94 (0.76)	6.7	3.5–9.8	0.19	0.13–0.31
MAS_MVC30%_	5.52 (1.31)	5.88 (1.53)	6.7	3.5–9.8	0.37	0.26–0.62
MAS_MVC45%_	8.28 (1.96)	8.81 (2.29)	6.7	3.5–9.8	0.56	0.39–0.94
MAS_MVC60%_	11.04 (2.61)	11.75 (3.05)	6.7	3.5–9.8	0.74	0.52–1.26
						
MAS_ABS_	6.5	6.5	\	\	\	\

Coefficients of reliability are also displayed.

MAS_MVC_  =  musculo-articular stiffness measured with a load corresponding to a percentage of maximal voluntary contraction; MAS_ABS_  =  musculo-articular stiffness measured with a constant absolute load; T1, T2  =  testing session number 1 and 2; CV  =  coefficient of variation; SEM  =  standard error of measurement; CL  =  confidence limits for CV and SEM.

**Table 2:** Summary of the frequency results obtained with a load corresponding to different percentages of maximal voluntary contraction, along with a constant load of 6.5 kg, over two testing sessions.

	Mean (SD) (s^−1^)		Reliability coefficients			
	T1	T2	CV (%)	95% CL	SEM (s^−1^)	95% CL
MAS_MVC15%_	1.615 (0.163)	1.622 (0.168)	4.8	2.8–6.7	0.060	0.040–0.114
MAS_MVC30%_	1.570 (0.162)	1.530 (0.179)	5.2	3.3–7.1	0.065	0.046–0.111
MAS_MVC45%_	1.407 (0.156)	1.414 (0.124)	6.4	3.9–8.8	0.071	0.051–0.118
MAS_MVC60%_	1.392 (0.093)	1.343 (0.116)	7.0	3.9–10.1	0.075	0.048–0.165
						
MAS_ABS_	1.462 (0.104)	1.442 (0.125)	3.3*	2.1–4.5	0.039	0.029–0.060

Coefficients of reliability are also displayed.

MAS_MVC_  =  musculo-articular stiffness measured with a load corresponding to a percentage of maximal voluntary contraction; MAS_ABS_  =  musculo-articular stiffness measured with a constant absolute load; T1, T2  =  testing session number 1 and 2; CV  =  coefficient of variation; SEM  =  standard error of measurement; CL  =  confidence limits for CV and SEM.

* =  significantly different from MAS_MVC60%_ (p<0.05).

**Table 3:** Summary of the musculo-articular stiffess results obtained with a load corresponding to different percentages of maximal voluntary contraction, along with a constant load of 6.5 kg, over two testing sessions.

	Mean (SD) (Nm^−1^)		Reliability coefficients			
	T1	T2	CV (%)	95% CL	SEM (Nm^−1^)	95% CL
MAS_MVC15%_	588.2 (147.6)	653.7 (179.6)	9.7	4.8–14.8	51.6	35.5–94.3
MAS_MVC30%_	889.3 (221.1)	859.8 (253.4)	8.1	3.4–12.7	60.3	43.2–99.4
MAS_MVC45%_	949.1 (182.8)	959.7 (207.2)	10.9	6.4–15.5	85.9	60.9–145.9
MAS_MVC60%_	1068.7 (276.2)	1016.9 (218.6)	12.6	6.3–18.8	98.8	63.7–217.6
						
MAS_ABS_	790.0 (127.9)	779.7 (126.3)	6.5*	4.1–8.9	40.2	29.7–62.6

Coefficients of reliability are also displayed.

MAS_MVC_  =  musculo-articular stiffness measured with a load corresponding to a percentage of maximal voluntary contraction; MAS_ABS_  =  musculo-articular stiffness measured with a constant absolute load; T1, T2  =  testing session number 1 and 2; CV  =  coefficient of variation; SEM  =  standard error of measurement; CL  =  confidence limits for CV and SEM.

* =  significantly different from MAS_MVC60%_ (p<0.05).

Further, the load CV (table 1) was slightly higher than the frequency CV (table 2) at low loads, whereas the trend was reversed at high loads (i.e. 60% of MVC).

## Discussion

This study quantified the contribution of variance in each component of MAS assessment. Although no statistical differences were detected between the two testing sessions for the variables examined, the current assessment procedures exhibited a CV of up to 12.6% for MAS. This magnitude of variability is certainly relevant when longitudinal changes in MAS are examined with reference to injury occurrence [15], [20], training [12] or fatigue [13]. Specifically, when conducting longitudinal or comparative research designs where stiffness is hypothesized to be influenced by a particular intervention, condition or timeframe, the identification of this variability dictates the required magnitude of change for a meaningful outcome. The level of variability in this study is slightly higher than that reported for different musculature which required different set-ups [4], [21]. This is probably due to the specific set-up needed for the assessment of the knee flexors, which is undoubtedly less comfortable for the participants than the assessment of the ankle flexors or the knee extensors.

The repeatability of the MVC measurement affects the assessment load added to the system, which consequently contributes variance to the MAS_%MVC_. Further, variability in the frequency of oscillation also affects the precision of MAS_%MVC_ results. Based on equation 4, it can be empirically proven that with the level of variability reported in this study (8 to 12.5%), CV_MAS_ is calculated as approximately the sum of CV_f_ and CV_m_, despite CV_f_ being multiplied by 2. The minor discrepancy between the reported results and the theoretical equation is likely due to the fact that the tables only report mean values which exhibit medium to large confidence limits. CV_MAS_ and CV_f_ exhibited a clear trend towards an increase as the assessment load increased and this could at least be partially explained as an impaired ability to maintain a stable position and the augmented physiological tremor when the test is performed at higher loads [22]. Further, Jaskolski et al [23] reported that repeated submaximal eccentric contractions (at 50% of MVC) caused an acute increase in tremor and contractile impairments in the elbow flexors. Such a response may have been present in the current study since the mechanical perturbations administered to generate the damped oscillations involved several eccentric contractions. It has to be acknowledged that despite the apparent trend CV_MAS_ and CV_f_ did not reach statistical significance and this can be attributed to the high inter-subject variability as expressed by the confidence limits for CV (table 2 and 3) and potentially to the sample size.

During MAS_ABS_ assessment, frequency was the only variable that could contribute to variance in equation 2. As depicted in equation 4, under such conditions the CV_MAS_ was calculated as the CV_f_ × 2. Interestingly, the use of a constant load yielded a very good level of reliability in frequency and MAS_ABS_ (tables 2 & 3), noticeably higher than that reported for the multiple load assessment and statistically significant when compared to a load corresponding to 60% of MVC. The lower variability can conceivably be attributed to the more standard conditions of testing administration for MAS_ABS_, which probably elicited more stable responses from the subjects. Further, the MAS_%MVC_ testing sessions were far longer and involved a number of measurements (MVC and MAS_%MVC_ with a range of loads). It can be advocated that fatigue may have increased the overall variability of MAS_MVC%_ compared to MVC_ABS_, as emerged in a recent examination of sub-maximal force up to 60% of MVC [24].

In conclusion, the assessment of MAS_%MVC_ is affected by a combination of variability in determination of the load applied and the frequency recorded, with approximately the same magnitude of contribution for each variable. In contrast, the MAS_ABS_ variability was consistently lower than MAS_%MVC_ variability. Based on the results presented, low and constant loads for MAS assessment yielded good to excellent levels of reliability and are thus ideal to implement in longitudinal research. This is particularly relevant in the clinical setting which typically uses one constant relatively low assessment load [25]. The assessment of MAS with multiple higher loads, which is more suitable for sporting situations [12], incorporates higher error. Such information can provide practitioners with a detailed understanding of the strengths and limitations of the methodology. This may require a compromise between the need to increase test specificity and the need to reduce the measurement error. The results obtained in the current study are particularly relevant for clinicians, physical therapists, conditioning coaches and sports scientists who are involved in athlete screening and physical development. The CV (in percentage) and the SEM (using the same units as the variable of interest) provide a useful measure of the expected trial-to-trial noise in the data, which is relatively unaffected by the inter-subject variability. Further, knowledge regarding the source and magnitude of error in assessment procedures can inform decision making regarding the usefulness of such screening procedures, including determination of appropriate sample size and minimum detectable change.
